# Teams in transition: Increasing role of advanced practice providers in inpatient antimicrobial use

**DOI:** 10.1017/ash.2023.266

**Published:** 2023-09-29

**Authors:** Reinaldo Perez, Michael Yarrington, Connor Deri, Michael Smith, Jillian Hayes, Rebekah Wrenn, Rebekah Moehring

## Abstract

**Background:** Antimicrobial stewardship strategies must be tailored to effectively engage prescribers with distinct training, experiences, and career paths. Advanced practice providers (APPs) have taken on increasing roles as primary team members in acute-care hospitals, but the impact of this practice shift on antimicrobial prescribing is unknown. We describe longitudinal trends in antimicrobial days of therapy (DOT) by attributed provider type in 3 hospitals. **Methods:** We performed a retrospective time-series analysis of antimicrobial use for the 7-year period of July 2015–June 2022 to investigate the changes by provider type at 3 hospitals: a major university hospital and 2 community hospitals. DOT, antibacterial, and antifungal agent groups were defined using National Healthcare Safety Network methods. We included anti-influenza and antiherpesvirus agents in the antiviral group. We defined protected agents as those targeted by hospital antimicrobial stewardship program policy (eg, requiring preauthorization). Provider type was defined by electronic health record user profiles in 3 categories: physician, trainees (residents, fellows and medical students), and APPs (nurse practitioners, physician assistants, and nurse anesthetists). We evaluated DOT per 1,000 days present over time by agent group to assess quarterly rate trends. Then, we calculated the percentage of total DOT by provider group. We used multinomial logistic regression to measure changes in percentage DOT across the clinician groups over time using physicians as the referent. **Results:** Across hospitals and provider groups, we observed an overall decrease in use rates for antibacterial and protected agents (17% each) and increased use rates for antiviral agents (38%) and antifungal agents (4%) (Table 1). Baseline distribution of DOT by provider group and change in distribution over time varied by hospital and agent group (Fig. and Table 2). The largest increases in percentage DOT attributed to APPs compared with physicians occurred in the university hospital with the following average increases per quarter: 1.5% for antibacterials, 3.9% for antivirals, 3.3% for antifungals, and 3.8% for protected agents (Table 3). Community hospitals had higher initial percentage DOT attributed to physicians, but both hospitals experienced increased percentage DOT attributed to APPs. Percentage DOT attributed to trainees varied significantly across agent groups and hospitals. **Conclusions:** Hospitals had differing baseline patterns of DOT attributed to provider groups, but all experienced increases in DOT attributed to APPs. APPs have increasing involvement in antimicrobial use decisions and should be engaged in future antimicrobial stewardship initiatives.

**Figure:**
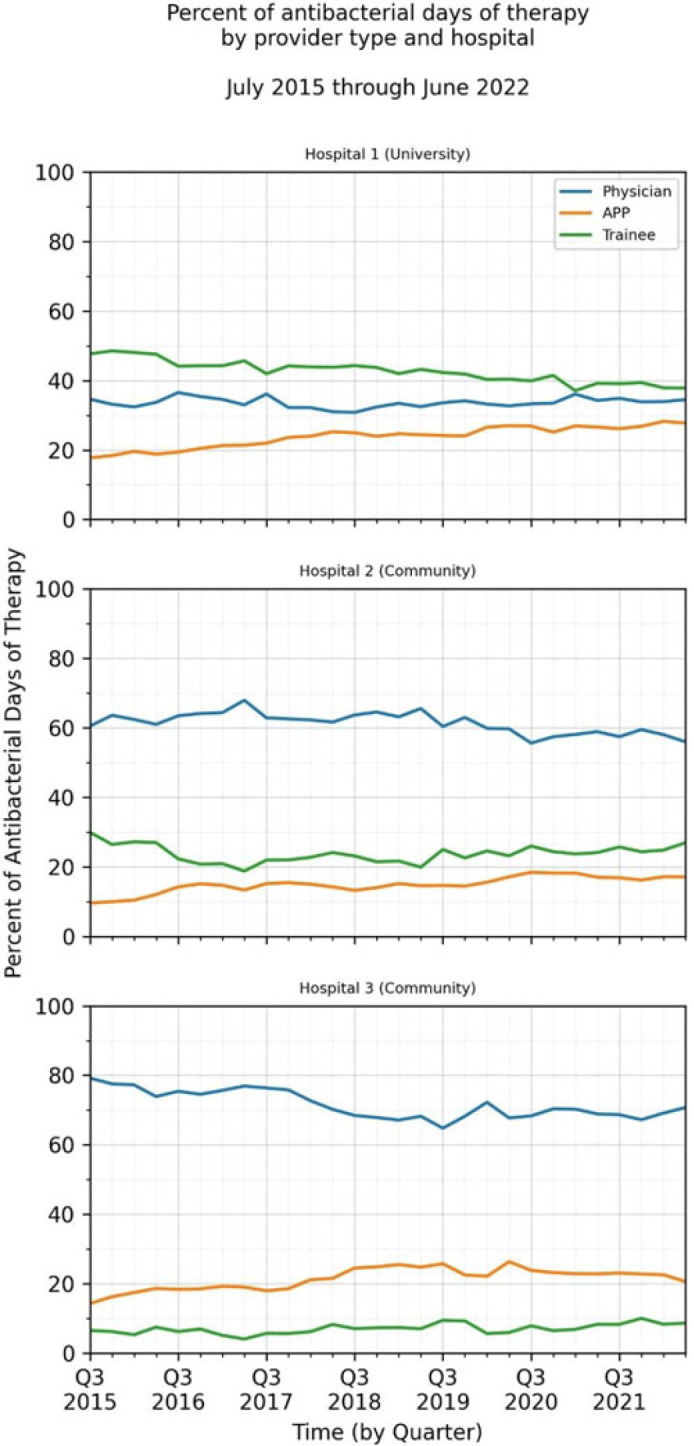

**Figure:**
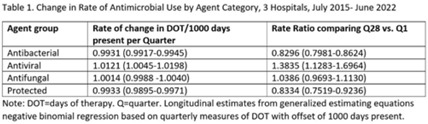

**Figure:**
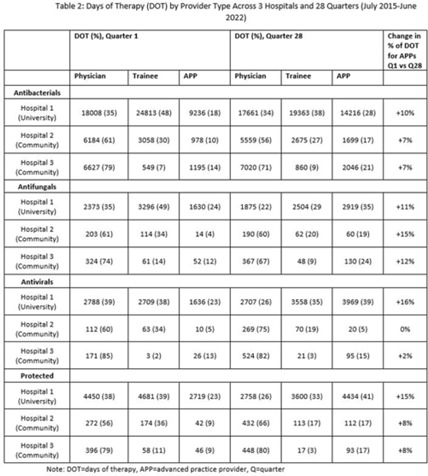

**Figure:**
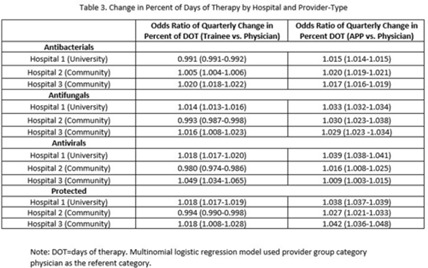

**Disclosures:** None

